# Coronary Atherosclerosis and Aortic Valve Disease as Long-Term Sequelae of Radiation Therapy in Childhood

**DOI:** 10.1155/2021/9324573

**Published:** 2021-11-30

**Authors:** Majken van den Handel Vestergaard, Ann Bovin, Erik Lerkevang Grove

**Affiliations:** ^1^Department of Cardiology, SLB Vejle, Denmark; ^2^Department of Internal Medicine, Faculty of Health, Aarhus University, Aarhus, Denmark; ^3^Department of Cardiology, Aarhus University Hospital, Palle Juul-Jensens Boulevard 99, 8200 Aarhus, Denmark

## Abstract

Coronary atherosclerosis and valvular heart disease are rare, but potentially severe sequelae following mediastinal radiation therapy. We present a case of premature ischemic heart disease and severe aortic stenosis in a 40-year-old woman following radiation therapy in childhood. We stress the awareness of prior mediastinal radiation therapy as an important risk factor for premature coronary atherosclerosis and valvular heart disease, particularly in younger patients without classical risk factors for coronary artery disease.

## 1. Learning Objective

Radiation therapy (RT) is an important risk factor for premature coronary atherosclerosis and valvular heart disease. Therefore, cardiopulmonary symptoms in patients with a history of RT should be carefully explored with, e.g., echocardiography and coronary CT, even in younger patients without any conventional risk factors for coronary artery disease.

## 2. Case Presentation

**Figure 1:**
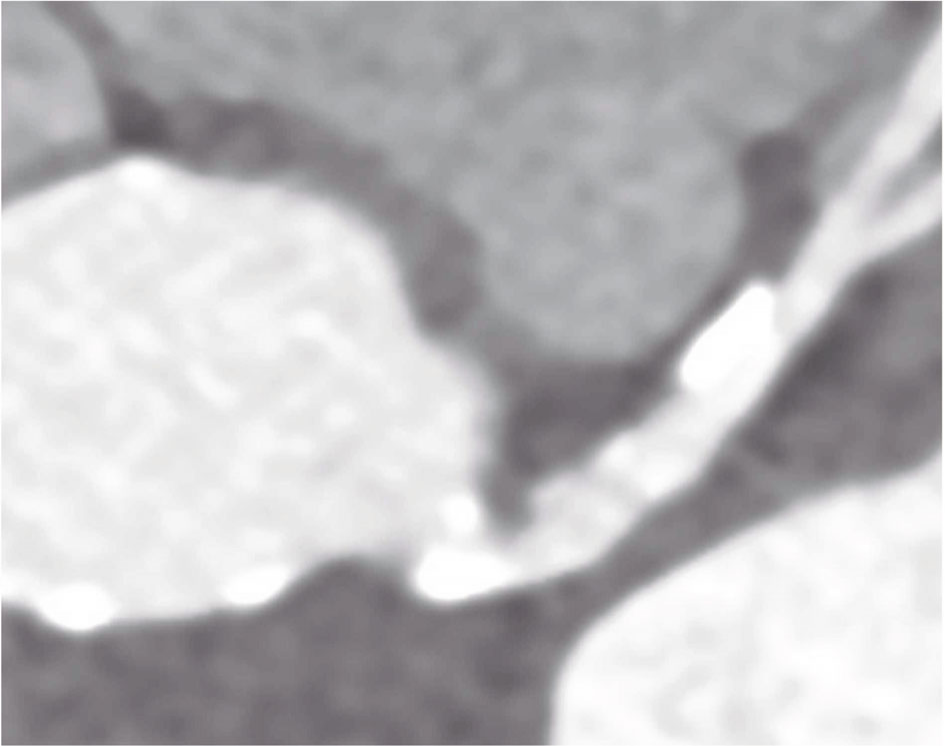
Computed tomography showing coronary atherosclerosis in the left main and the left anterior descending artery.

A 40-year-old woman with prior mediastinal radiation therapy (RT) due to lung cancer at the age of 3 presented with recurrent syncope during exercise. During most of her adulthood, she had felt a reduced physical capacity compared to her peers. Echocardiography revealed moderate stenosis and insufficiency of a tricuspid aortic valve. She had no classical risk factors for development of coronary artery disease: no history of smoking, no diabetes, normal cholesterol levels, no arterial hypertension, and no family history of premature ischemic heart disease. Initial assessment consisted of exercise testing and coronary CT angiography. Exercise testing resulted in dizziness and significant ST-segment depression of 2-3 mm in inferior and lateral leads, but no chest pain. CT angiography revealed severe stenosis of the left main coronary artery (Figure 1) and ostial stenosis of the right coronary artery (RCA), confirmed by coronary angiography (CAG). Following a joint cardiology-cardiothoracic multidisciplinary team meeting (MDT), percutaneous coronary intervention (PCI) with drug eluting stents was performed in the left main coronary artery and ostially in the RCA.

A few months later, ischemia was revealed during routine exercise testing as part of the outpatient cardiac rehabilitation program. A new CAG revealed stent occlusion ostially in the RCA. Re-PCI with stenting was performed; however, symptoms persisted, and re-echocardiography now revealed progression to significant aortic valve stenosis and significant insufficiency. Due to prior mediastinal RT and excessive aortic calcification and fibrosis as well as narrow diameter of both the aorta ascendens (annulus 23 × 17 mm and sinutubular junction 20 × 21 mm), descendens, and arcus, transcatheter aortic valve implantation (TAVI) was recommended by consecutive MDT meetings. TAVI was performed by inserting a 20 mm Sapien 3 valve via femoral approach without complications, along with redilatation of the RCA. She was asymptomatic at three years follow-up after TAVI.

Severe coronary atherosclerosis and valvular heart disease have been reported as rare sequelae following mediastinal RT [1]. RT-induced coronary artery disease is associated with a characteristic pattern: primarily proximal stenosis, typically located at the ostium of the RCA and the left main coronary artery [2]. In RT-induced valvular heart disease, left-sided heart valves are more commonly affected [3] and patients with prior mediastinal RT and severe aortic stenosis have significantly reduced long-term survival compared to patients with aortic stenosis but without prior RT [4]. Adults treated with RT in childhood may present with cardiac disorders as late as 30 years after therapy, and cardiac screening is therefore important [5]. Suggestions for a screening program containing echocardiogram and transesophageal echocardiography 10 years postradiation and every 5 years are available, but no standardized long-term screening program exists [6]. Thus, we stress caution and awareness of symptomatic patients with prior mediastinal RT due to an increased risk of severe coronary atherosclerosis and valvular heart disease even among younger adults without conventional cardiovascular risk factors.

## Data Availability

Data are available upon reasonable request to the corresponding author.
